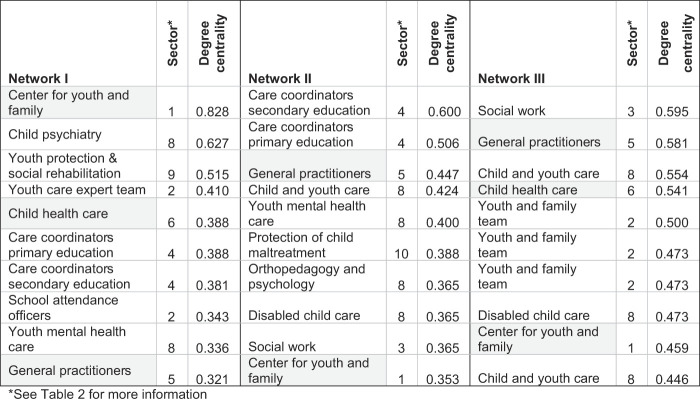# Cross-sectoral collaboration: comparing complex child service delivery systems

**DOI:** 10.1108/JHOM-07-2021-0281

**Published:** 2022-03-08

**Authors:** Mariëlle Blanken, Jolanda Mathijssen, Chijs van Nieuwenhuizen, Jörg Raab, Hans van Oers

**Affiliations:** Tranzo, Scientific Center for Care and Wellbeing , Tilburg University , Tilburg, The Netherlands; Department of Organization Studies, School of Social and Behavioral Sciences , Tilburg University , Tilburg, The Netherlands

**Keywords:** Child service delivery system, Social network analysis, Cross-sectoral collaboration, Organizational networks, Client referral, Differentiation, Integration

## Abstract

**Purpose:**

To help ensure that children with social and behavioral health problems get the support services they need, organizations collaborate in cross-sectoral networks. In this article, the authors explore and compare the structure of these complex child service delivery networks in terms of differentiation (composition) and integration (interconnection). In particular, the authors investigate the structure of client referral and identify which organizations are most prominent within that network structure and could therefore fulfill a coordinating role.

**Design/methodology/approach:**

The authors used a comparative case study approach and social network analysis on three interorganizational networks consisting of 65 to 135 organizations within the Dutch child service delivery system. Semi-structured interviews with the network managers were conducted, and an online questionnaire was sent out to the representatives of all network members.

**Findings:**

The networks are similarly differentiated into 11 sectors with various tasks. Remarkably, network members have contact with an average of 20–26 organizations, which is a fairly high number to be handled successfully. In terms of integration, the authors found a striking diversity in the structures of client referral and not all organizations with a gatekeeper task hold central positions.

**Originality/value:**

Due to the scarcity of comparative whole network research in the field, the strength of this study is a deeper understanding of the differentiation and integration of complex child service delivery systems. These insights are crucial in order to deliver needed services and to minimize service silos and fragmentation.

## Introduction

To meet the varied needs of children and youth with social and behavioral health problems, collaboration between service delivery organizations for child welfare and mental health is considered vital ( Brown *et al.* , 2014 ; Bunger, 2013 ; Bunger *et al.* , 2014 ; Bunger and Huang, 2019 ; Bustos, 2020 ; Colvin, 2017 ; Colvin and Miller, 2020 ). For comprehensive, tailor-made and seamless service delivery, it is critical that these organizations coordinate services by timely and appropriate referring clients and sharing information or staff expertise with one another ( Bolland and Wilson, 1994 ; Bunger *et al.* , 2014 ; Sowa, 2009 ). To help ensure that children get the support services they need from professionals with the required skills, child service delivery organizations collaborate in cross-sectoral networks ( Brown *et al.* , 2014 ; Colvin and Miller, 2020 ). These networks consist of a broad range of actors, such as mental healthcare, education, childcare and nursery, safety, protection and social rehabilitation, specialized youth care, community service and social support. The importance of network collaboration for the success of a service delivery system is well-established in the public administration literature ( Isett *et al.* , 2011 ; Kramer, 2014 ; Provan and Lemaire, 2012 ; Provan and Milward, 1995 , 2001 ; Raab *et al.* , 2015 ; Smith, 2020 ; Turrini *et al.* , 2010 ).

Despite these insights, service fragmentation and service silos remain persistent problems in the field of health and human services, including child and youth services ( Bustos, 2020 ; Cooper *et al.* , 2016 ; Nicaise *et al.* , 2013 ; Provan and Huang, 2012 ). If organizations are reluctant to share resources or information (leading to service silos), and there is a lack of coordination or collaboration within the child welfare and healthcare service delivery system (leading to service fragmentation), the risk is considerable that children and youth in need do not get the right service at the right time or even will be overlooked and left untreated ( Bustos, 2020 ). However, networks are no panacea and can also fail ( Kenis and Raab, 2020 ). Understanding their set-up and structure is therefore crucial for the effective delivery of child and youth services.

Unfortunately, there is little empirical knowledge of cross-sectoral service delivery networks pertaining to child and youth services. Due to their multidisciplinary nature, it is largely unclear how these networks are composed (network differentiation) and how the organizations within a network are interconnected (network integration) ( Bustos, 2020 ; Colvin, 2017 ; Colvin and Miller, 2020 ). From the organization design theory perspective, both differentiation and integration are fundamental and interlinked issues relevant for the functioning of an interorganizational network ( Kenis and Raab, 2020 ). The differentiation of a network determines the division of tasks within the network consisting of a variety of organizations with access to diverse expertise and resources, and the integration reveals patterns of collaboration between those different organizations. In order to deliver needed services timely and appropriate, to minimize service silos, service fragmentation and duplication of services and to facilitate more informed decision-making processes, more information about the structure of child welfare and healthcare service delivery systems in terms of differentiation and integration is indispensable for network managers ( Provan and Milward, 1995 , 2001 ; Smith, 2020 ).

Client referral is one of the key processes in the network to ensure that the needed support services are provided timely and appropriate ( Brown *et al.* , 2014 ; Colvin and Miller, 2020 ). To be able to refer clients between organizations in the network in a proper way, the child service delivery organizations with a gatekeeper function are core organizations and need to have a central position in the network. Since the role of core organizations is critical for network success ( Provan and Milward, 2001 ), it is relevant to assess whether the likely core organizations regarding client referral indeed have a central position in the network and are therefore able to fulfill a coordinating role. This mechanism, referred to in the literature as *selective integration* , means “that network links must be targeted and appropriate, so that those organizations that need to work closely together do so, while others do not” ( Provan and Lemaire, 2012 , p. 644).

Therefore, this study explores and compares the structure of three complex child welfare and healthcare service delivery networks in terms of differentiation and integration. In particular, we investigate the structure of client referral and identify which organizations are most prominent within that network structure, and which could therefore fulfill a coordinating role. By studying the differentiation of the networks, we gain a better understanding of the various participating organizations and sectors and the extent to which the networks are consistent regarding their composition and task division. By examining and comparing the integration of the networks, we gain more insight in which organizations and sectors do and do not collaborate, and whether organizations with a gatekeeper function are able to refer clients between organizations in the network due to their structural position in the network. The whole network approach of our study meets the call for a complex systems approach in combination with social network analysis to examine the functioning of the network as a whole, and especially in the field of child and youth services ( Benham-Hutchins and Clancy, 2010 ; Colvin and Miller, 2020 ; Kitson *et al.* , 2018 ; Mischen and Jackson, 2008 ; Morçöl and Wachhaus, 2009 ; Quinn *et al.* , 2012 ; Stevens and Cox, 2008 ; Stevens and Hassett, 2007 ). Indeed, by examining the multilateral relations rather than focusing on individual organizations and their direct relations only, it is possible to understand how processes such as client referral generate collective outcomes ( Berthod *et al.* , 2017 ; Colvin and Miller, 2020 ; Provan *et al.* , 2007 ; Van der Ham *et al.* , 2020 ).

## Methods

### Research setting

The research field of this study is the societal and administrative context of the Dutch child and youth service delivery system. Like many countries ( Abimbola *et al.* , 2019 ; Anttonen *et al.* , 2003 ; Jiménez-Rubio and García-Gómez, 2017 ; Muñoz *et al.* , 2017 ; Sellers and Lidström, 2007 ; Senkubuge *et al.* , 2014 ), the Netherlands recently implemented welfare and healthcare state reforms that shifted key responsibilities for the welfare and healthcare system from the central to local levels of government. The reform began with the introduction of the Dutch Social Support Act in 2007 ( Dijkhoff, 2014 ; Putters *et al.* , 2010 ), followed by the decentralization of the Child and Youth service delivery system by shifting responsibilities from the national and regional governments to the local governments in 2015 (Child and Youth Act, 2014). Since then, municipalities have become fully responsible for the child welfare and healthcare service delivery system.

In this study, a comparative case study ( Collins *et al.* , 2007 ; Swanborn, 2010 ) was conducted of three interorganizational networks of child and youth services in different-sized municipalities in the Netherlands. Network I is located in a midsize municipality (around 180,000 citizens), Network II is located in a small municipality (around 66,000 citizens) and Network III covers four very small municipalities that collaborate in providing child and youth services (with 13,000–20,000 citizens per municipality, i.e. a total of about 60,000 citizens).

#### Research population and boundary specification

The research population consisted of organizations that participated in the child and youth service delivery networks, i.e. network members, with the representatives of the network members as the units of observation ( Wasserman and Faust, 1994 ). The following definition of a network was used: *the network of child and youth services consists of organizations with whom the local government, according to the network manager, works together to achieve the main network goal of the Child and Youth Act* . Employees who act as boundary spanners between their organizations in the network were the respondents ( Kramer, 2014 ; Williams, 2002 ). The network managers – the responsible managers of the municipalities’ child and youth support departments – were asked to identify the network members and to select the boundary spanners for each network. Network members were included when they met the abovementioned definition of the network. The selection of network members, including boundary spanners, was checked by colleagues of the municipalities’ child and youth support department, and were compared to information on network members from the administrative system of the department. There was no disagreement concerning the selection of network members including boundary spanners. We thus applied a combination of the nominalist and realist approach to network boundary specification in as we first nominally defined a criterion to include organizations and then used the judgment of participating individuals in the network to determine the boundaries ( Laumann *et al.* , 1989 ).

Since the individual professionals of some network members operated within a limited working area – such as school care coordinators in education organizations, school attendance officers in municipal organizations, general practitioners (family doctors) and organizations for childcare and nursery – we invited more than one boundary spanner from these network members for the survey. For example, in Network I, there were a total of thirty general practitioners in the municipality. As the working area of one general practitioner was limited to a small part of the municipality, we invited them all to participate in the research. Since the organization level is the level of data analysis, we aggregated the results for these boundary spanners to the level of their organizations or professional group (see data analysis for information on the applied rules).

For Network I, we also used a threshold for the selection of network members from the sector “specialized youth care organizations”. As a relatively large number of these organizations only had a few juveniles in treatment in one year and therefore had peripheral positions in the network, we selected only the organizations that had a minimum of six juveniles receiving care in 2017 (94 of 162 organizations). This threshold is generally used for privacy reasons. However, although the focus is on the relationships and not the individual persons, it is still a low number, and the relationships are heavily influenced by individual cases. The final selection of 94 specialized care organizations together accounted for 98% of all juveniles residing in that municipality and receiving specialized care in the year 2017. In this way, we were able to strike a balance between a questionnaire that is manageable for the respondents and yields representative information about the specialized youth care organizations. Table 1 displays the number of network members, including the response rates.

### Data collection

The data of the three networks were collected in the period of November 2017 to September 2018 and consisted of two steps. First, semi-structured interviews with the network managers were conducted. The aim of the interviews was to identify the boundaries of the network by determining the network members, their main tasks and categorizing them into different sectors and to select representatives of the network members as potential respondents for the online questionnaire. Second, an online questionnaire was sent out to the representatives of all the network members to collect data about the relations between the organizations. In the questionnaire, to measure the number of all contacts between the organizations, the respondents were presented a list of all the organizations of the network and were asked to identify the organizations with which their organization had contact at least once a year, including face-to-face contact (meeting, consultation, conference), by telephone or e-mail. To measure client referral relations between the organizations, the respondents were also asked to indicate whether their organization had contact with the other organizations regarding client referral.

### Measures

Network structure refers to patterns of relationships that exist within a given boundary ( Wasserman and Faust, 1994 ). It consists of nodes (organizations) that compose the network, ties that connect the nodes, and the patterns, structures and nature of the relationships that result from these connections ( Popp *et al.* , 2014 ). To explore and compare the structure of complex child welfare and healthcare service delivery networks and moreover the structure for client referral, the concepts of differentiation and integration were measured.

#### Network differentiation

The structural network characteristics size, tasks and sectors were used to describe and compare the differentiation of the networks ( Popp *et al.* , 2014 ; Provan and Lemaire, 2012 ; Shortell *et al.* , 2015 ; Smith, 2020 ). During the interview, the network manager selected the participating organizations according to the definition: *organizations with whom the local government works together to achieve the main network goal of the child and youth act* . Then, the network manager was asked to classify the organizations into sectors and to describe the main task of the organization.

#### Network integration

Network integration was measured by the number of “active organizations”, “isolates”, “ties”, “density”, “average degree centrality” and “centralization” of the networks for both *all contacts* and *client referral contacts* . *Number of active organizations* is the total of organizations connected to another organization in a network; the *number of isolates* is the total of organizations not connected to another organization in the network; and the *number of ties (relations)* is the total of ties that is present in a network. *Density* refers to how cohesive a network is, computing the number of ties in a network, divided by the maximum number of ties that are possible ( Kilduff and Brass, 2010 ). The higher the score (ranging from 0 to 1), the more relations between organizations are present in the network ( Colvin, 2017 ). *Average degree centrality* is the average number of connections per organization in the network ( Scott and Carrington, 2011 ). *Centralization* refers to the power and control structure of the network and reveals whether network links and activities are organized around any particular single organization or small group of organizations ( Kilduff and Brass, 2010 ; Provan *et al.* , 2009 ; Provan and Milward, 1995 ; Scott and Carrington, 2011 ). Scores range between 0 and 1, with 1 being the highest possible centralization.

Beside the abovementioned network integration measures, to identify the organizations that are most prominent within the client referral structure, we calculated *degree centrality* . Degree centrality computes the number of other organizations to which a specific organization in the network is connected ( Scott, 2011 ).

### Data analysis

To calculate measures that describe the structure in terms of network differentiation and integration, we used Excel, Ucinet ( Borgatti *et al.* , 2002 ) and Visone ( Brandes and Wagner, 2004 ). The latter was mainly used to visualize the network graphs of the client referral structure. In Excel, the relational data (contact and client referral) were converted into adjacency matrices that were then inserted in Ucinet. To reflect relationships reported by each organizational dyad and in that way capturing “any link”, the networks were “symmetrized” ( Provan *et al.* , 2010 ). This method examines “unconfirmed” or unidirectional network ties, which are ties where a respondent identifies a link between their own and another organization, but the other organization does not confirm that collaboration exists ( Provan *et al.* , 2010 , pp. 3050-3051). We applied the following rule to create the adjacency matrices: a relation between two network members was coded as existing if at least one of the (boundary spanners of the) network members indicated this relation. The missing values were entered as a reciprocal relationship per responding organization (i.e. transposing the column in an adjacency matrix with the corresponding missing rows). This method is known as the procedure of labeled reconstruction ( Stork and Richards, 1992 ) to manage nonresponse. Then, in Ucinet, we computed the multiple network measures (number of active organizations, isolates, ties, density, centralization and average degree centrality) and degree centrality per network. Subsequently, we aggregated the adjacency matrices of client referral to the sectoral level in Excel. We used a fourfold division for the relations between the sectors. If 0–20% of all possible ties were present, we coded 0 (no relation). If we found between 20–40%, 40–60% or at least 60% of all possible ties present, we coded, respectively, 1 (weak connection), 2 (average connection) and 3 (strong connection). Finally, in Visone, we inserted the aggregated adjacency matrices of client referral to visualize the graphs of the client-referral networks. In the graph, we used different widths and color to show the connection strength of ties between the sectors.

## Results

### Network differentiation

Network I, with 135 participating organizations, is the largest network compared to Network II with 86 and Network III with 75 organizations. The networks are composed of organizations from various sectors performing different tasks ( Table 2 ).

Organizations that exchange (early warning) signals of support needs by children, youth and families with other organizations in the network have a signaling task. Gatekeepers are organizations that are legally authorized to refer clients to child and youth services covered by the Child and Youth Act. Organizations tasked with providing services deliver various child and youth support and care services. All the sectors from Table 2 are present in the networks, with the exception of volunteer organizations in Network II since they were not designated as network members by the municipality.

### Network integration

Table 3 presents the results regarding the integration of the networks. All the organizations of the different networks have a relation based on at least one type of tie with at least one other organization in their network, i.e. there are no isolates. The number of ties in Network I (3,368 ties) is the largest compared to Network II (1728 ties) and Network III (1950 ties). Network III shows the highest density of the three networks (0.351). In other words, approximately 35% of all possible ties in Network III exist. For Networks II and I, this figure is about 24 and 19%, respectively. Organizations in Network I have an average degree centrality of 25 organizations. For organizations in Networks II and III, the figure is, respectively, 20 and 26 organizations. The centralization scores of the three networks vary slightly. On a scale from 0 to 1, with 1 being the highest possible centralization, centralization scores of around 0.6 indicate that the ties in each network are organized around one central or a few central organizations.

### Client referral network structure

**Figure 1:**
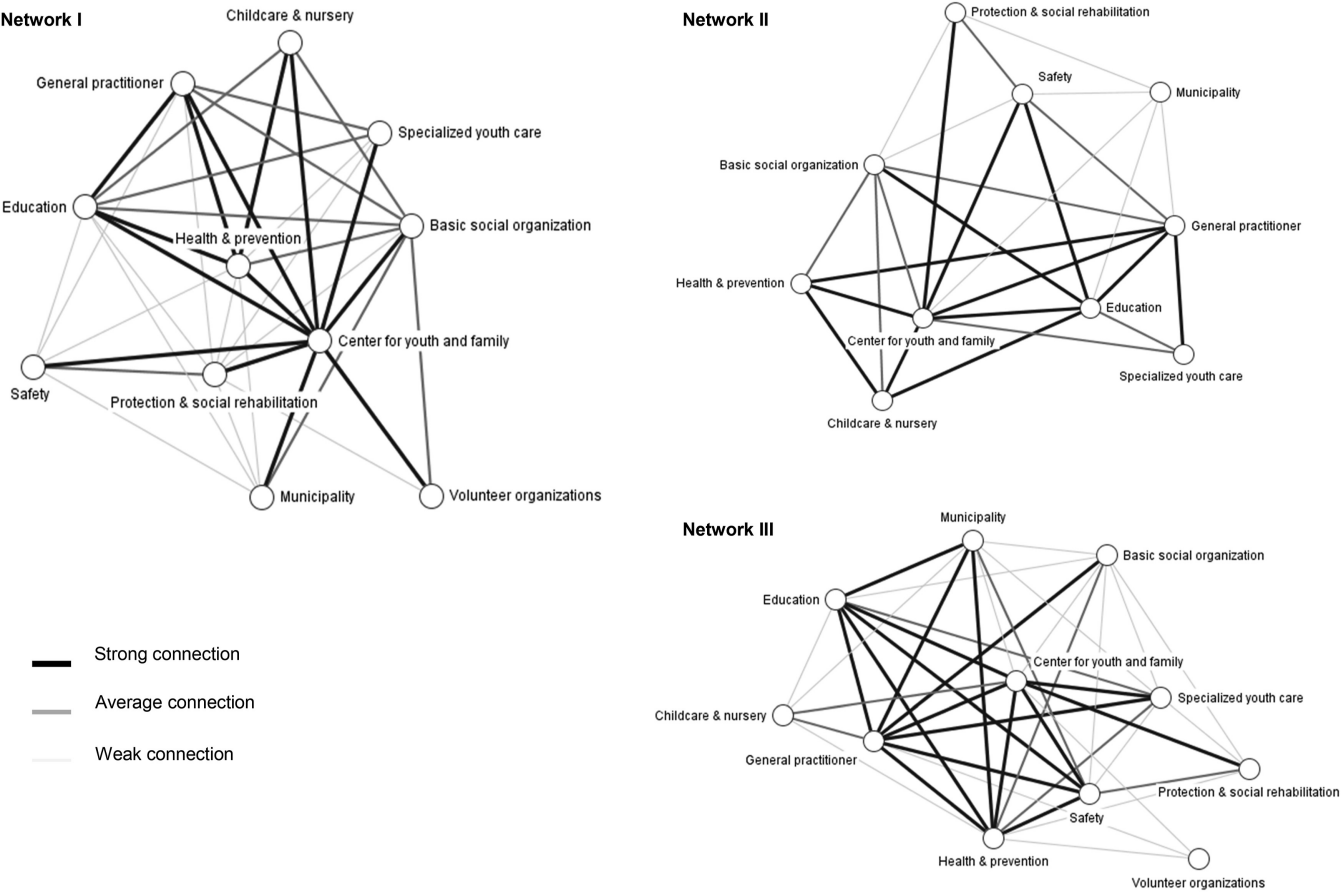
Interconnectedness of the client-referral networks based on connection strength between sectors in the network

The structure of the networks – regarding client referral – at the sector level shows that not every sector is connected to all others. Also, the connection strength differs between the sectors. Figure 1 presents the network diagrams of the client referral networks at the sector level. The different width and shade of the ties show the connection strength between the sectors. Comparing the three networks in Figure 1 shows that Network II has less relationship (ties) based on client referral between the different sectors than Networks I and III. Further, the sectors “Center for youth and family”, “Education”, “General practitioner” and “Health and prevention” have many relationships based on client referrals with other sectors in the network, and there is often a strong connection, except for the sector “Health and prevention” in Network II.

Table 4 shows the differentiation and integration results of the networks based on client referral. The vast majority of the organizations refer clients among each other (92–98% active organizations). The isolates in Networks I and II were specialized youth care organizations with a few juveniles in treatment (less than 15 clients), and in Network III, these concerned four volunteer organizations, one specialized youth care organization and one safety organization. It applies for each network that less than 20% of all possible ties were present. The organizations in Network I, II and III were found to have contact with, respectively, an average of approximately 16, 12 and 14 organizations. The centralization score of network I (0.723) is higher than the scores of Networks II (0.471) and III (0.415).

Table 5 shows, per network, the 10 organizations holding the most central position based on their degree centrality. In every network, the (gray-marked) organizations with the task of gatekeeper (center for youth and family, general practitioners and child healthcare) are among the most prominent organizations in the networks, except for child healthcare in Network II. In Network I, the center for youth and family holds the most central position; in Network II and III, this organization is less prominent, in these networks respectively care coordinators and social work hold the most central position. Compared to Network I, in Networks II and III, the general practitioners have a central position. Child healthcare has a relatively central position in Networks I and III.

## Discussion and conclusion

This study examined the structure of three complex child welfare and healthcare service delivery networks in terms of differentiation and integration. Differentiation and integration are both necessary conditions to successfully deal with complex family issues. Differentiation is needed to address problems that are too complex, expensive or persistent for one organization or government to handle on its own ( Agranoff and McGuire, 2001 ; O’Toole Jr, 1997 ; Provan and Lemaire, 2012 ), and network integration is required to effectively achieve the network goals ( Provan and Milward, 1995 ; Raab *et al.* , 2015 ; Smith, 2020 ). Client referral is one of the core processes in the network to ensure that the support services that children need are provided ( Brown *et al.* , 2014 ; Colvin and Miller, 2020 ), for that reason, we studied the structure for client referral in particular.

The three studied networks are relatively comparable in terms of differentiation. There is a differentiation into 11 sectors and various tasks (gatekeeper, signaling and providing services) among network members, which emphasizes the importance of understanding the functioning of networks as a whole. Even though Network I has more network members, it spans an equal number of sectors as the other networks. This is not surprising, since the networks are embedded in the same institutional framework of the Child and Youth Act.

In terms of integration, we found that the smallest network (Network III) is denser than the other two, as expected, since density scores are sensitive to network size ( Borgatti *et al.* , 2018 ). Therefore, in order to provide a more digestible understanding of density, we measured also the average number of connections per organization in the network (average degree centrality). Remarkably, in each network, organizations have contact with an average of 20–26 organizations, which is a fairly high number to be handled successfully and effectively. It is known that most organizations tend to have limited numbers of ties (or at least strong ties), as social actors have limited resources, energy, time and cognitive capacity, and cannot maintain large numbers of strong ties ( Hanneman and Riddle, 2005 ).

These findings regarding the interlinked concepts of differentiation and integration are relevant in the light of the aim of welfare and healthcare state reforms. The major decentralization of the Dutch child and youth service delivery system was meant to facilitate integrated care in families’ own environment by the decompartmentalization of budgets and the local responsibility to organize child welfare and healthcare ( Boogers and Reussing, 2019 ; Boogers *et al.* , 2009 ; Bosscher, 2012 ; Nooteboom, 2021 ). The diversity and overall connectedness of the networks show that the desired variation of sectors with access to diverse expertise and resources, and the division of tasks (gatekeeper, signaling and providing services) are present within the networks, which is a critical condition for integrated care ( Nooteboom, 2021 ). On the other hand, our findings demonstrate the potential risk of inefficient and ineffective functioning of (parts of) the network due to a high number of relations that need to be maintained by its members. In order to make relations more targeted, it is important that network managers consider and investigate how key processes such as information sharing, client referral and administrative processes can be structured within the network effectively ( Kenis and Raab, 2020 ; Provan and Lemaire, 2012 ).

When we compare the structures for client referral between the networks, again the networks are relatively comparable in terms of differentiation. Regarding integration, the networks based on client referral are not connected as a whole, because there are 3–6 isolates per network. These isolates turn out to be peripheral organizations mainly tasked with providing services, predominantly highly specialized youth care. Moreover, there was a striking diversity in the structure of the client referral networks. At the sectoral level, the integration client referral of Network II is different compared to the other two networks. Overall, Network II has fewer relationships (ties) based on client referral, and the expected core organizations of the sector “Health and Prevention” do not have many relationships or a strong connection with other sectors in the network. Furthermore, at the level of organizations, we found that Network I (centralization 0.723) is more centrally integrated than Networks II (centralization 0.471) and III (centralization 0.415). In Network I, client referral is primarily organized around the center for youth and family (degree centrality 0.828). In Networks II and III, client referral is less centrally organized and the organizations with a gatekeeper task do not hold central positions. Instead, education and basic social organizations are the most prominent, as they had contact regarding client referral with the greatest number of other organizations in the network. This could mean that, in Networks II and III, it is not the expected core organization that fulfills the main coordinating role regarding client referral – i.e. the center for youth and family – but organizations tasked with signaling and providing services such as social work or school care coordinators.

At the core of the decentralized Dutch child and youth service delivery system are the locally formed centers for youth and family ( Nooteboom, 2021 ). These centers, as front office of the municipality, are the linking pin between preventive support (e.g. basic care and universal pedagogical provisions) and primary care (e.g. child healthcare, general social work, parenting support) and specialized care (e.g. youth care services, specialized mental healthcare, child protection, high intensive psychiatric support, residential youth care) ( Bosscher, 2012 ). To be able to refer clients between organizations in the network in a proper way, these linking pin organization need to have a central position regarding client referral in the network. In this respect, Network I operates in a more targeted manner than Networks II and III, which could imply that children and youth residing in one municipality are at a greater risk of being overlooked and left untreated than in other municipalities. There are numerous possible explanations for the found differences regarding integration. In general, there is a consistency between the level of trust and the functioning of a network as a whole ( Klijn *et al.* , 2010 ; Zaheer *et al.* , 1998 ). Higher levels of trust are associated with increased performance, efficiency or satisfaction for one or more parties in interorganizational relationships ( Zaheer *et al.* , 1998 ). More specifically, trust has been found to reduce transaction costs and to increase inner network stability, commitment and information sharing ( Dyer and Chu, 2003 ; Klijn *et al.* , 2010 ; Kramer, 2014 ; Nielsen and Nielsen, 2009 ; Nooteboom, Berger and Noorderhaven, 1997 ). Perhaps the level of trust was higher in Network I compared to Networks II and III. Moreover, the current state of development of the network can be a possible explanation for the found differences in integration. As a network system matures over time, relationships may become more cemented and robust ( Ahuja *et al.* , 2012 ; Provan *et al.* , 2009 ). Such stability of network relationships turns out to be a major factor in explaining network effectiveness regarding client services ( Provan and Milward, 1995 ). Maybe, Networks II and III needed more than three years to regroup after a major shakeup like a decentralization of the child welfare and healthcare system: a period previously indicated as sufficient time for networks to stabilize ( Raab *et al.* , 2015 ).

For this study, some methodological remarks can be made. First, the network boundaries were determined by the respective network managers of the municipalities. All organizations partnered by a local government to achieve the main network goal of the Child and Youth Act were included. However, it could well be that there are other organizations that contribute to the network goal that do not collaborate with the local government but only with other members of the network. Nevertheless, we chose this strict determination since the application of this clear criterion makes it easier to reproduce the results ( Provan *et al.* , 2007 ). Second, the results must be seen in the specific institutional context. The networks are not fully mandated networks, but they have a strong institutional component due to the authorization of the gatekeepers to commission child and youth services covered by the Child and Youth Act. As a result, the differentiation of the networks can hardly differ between the municipalities. In contrast, the integration of the networks can certainly differ, as the network managers have the opportunity to structure the relationships within the network. Third, the static character of network analysis should be recognized, and because networks are not static but dynamic systems, the results should be interpretated with caution ( Human and Provan, 2000 ; Lemaire *et al.* , 2019 ; Popp *et al.* , 2014 ). Fourth, although we were able to determine and compare the differentiation and integration of the child welfare and healthcare services delivery networks that are critical for network success ( Kramer, 2014 ; Popp *et al.* , 2014 ; Provan and Lemaire, 2012 ; Provan and Milward, 1995 , 2001 ; Smith, 2020 ; Turrini *et al.* , 2010 ), we did not examine whether the structural form has an actual influence on network outputs or outcomes. Fifth, as whole network data allows for very powerful descriptions and analyses of social structures, we used the whole network approach which yields the maximum of information ( Hanneman and Riddle, 2005 ). This means that the networks were “symmetrized” in order to reflect relationships reported by each organizational dyad and capturing “any link” ( Provan *et al.* , 2010 , pp. 350–351). However, as this approach examines unconfirmed ties, it may have led to an overestimation of some network ties, especially for the nonresponse organizations. Fortunately, with the exception of the general practitioners, all the expected core network members responded. Most of the nonresponders were network members at the periphery of the network, such as the municipality’s department of safety, organizations for childcare and nursery or organizations for youth protection and social rehabilitation. Finally, there are other centrality measures, such as betweenness centrality and closeness centrality, which could have been used to identify the organizations that are most prominent within the client referral structure. However, we have chosen for degree centrality for several reasons. First, the data are undirected, and therefore, actors differ from one another only in how many connections they have ( Hanneman and Riddle, 2005 ). Second, we chose degree centrality, because of the relatively high amount of missing data with response rates between 52% and 68%. Degree centrality is a local centrality measure and therefore less sensitive to missing data, compared to global centrality measures. Third, closeness centrality scores are meaningless for disconnected networks (with at least one network isolate) such as ours, as the paths from all the other nodes to the isolates are infinitely long ( Wasserman and Faust, 1994 ).

For further research, we believe it is relevant to study how service delivery systems operate during the different development stages of a network. As a network system evolves over time, knowledge and information about network members and their tasks, especially regarding core organizations, will spread and the network structure will become more established ( Provan *et al.* , 2009 ). This could include outputs such as offering well-coordinated child and youth services geared to local and individual situations and needs, working on the basis of integrated policies, achieving an overall cost reduction for the municipalities ( Bosscher, 2012 ) or even the (enhanced) well-being of children and young adults ( Provan and Milward, 1995 , 2001 ). In addition, research could explore whether there is a minimum–maximum range on the degree of differentiation and the efforts to achieve integration for an effective functioning of the network, also known as the unity-diversity tension described by Saz-Carranza and Ospina (2011 ).

Due to the scarcity of comparative whole network research in the field and despite the limitations, the strength of this study is a deeper understanding of the differentiation and integration of complex child welfare and healthcare service delivery systems. The study provides empirical evidence of multidisciplinary and interorganizational interdependencies that are often assumed in this field but have rarely been demonstrated to exist through systematic empirical analysis. The observed differentiation of the networks, demonstrated by the multitude and heterogeneity of sectors and organizations, supports a conception of child welfare and healthcare practice as a complex service delivery system ( Colvin and Miller, 2020 ). At the same time, the wide span of the networks emphasizes the importance of targeted and appropriate links between organizations, i.e. selective integration ( Provan and Lemaire, 2012 ). Network managers should realize that a larger and/or more diverse network with a broader division of labor demands attention, time and resources to achieve the integration necessary to successfully accomplish the shared goals of the network ( Kenis and Raab, 2020 ).

## Figures and Tables

**Table 1:** Summary of research population and response

	Network I*	Network II*	Network III*
	2018	2018	2018
Number of invited network members	135	86	75
Number of responding network members	70	49	51
Response percentage network members	52%	57%	68%

**Note(s):** * Network I in municipality with around 180,000 citizens, Network II in municipality with around 66,000 citizens and Network III in four municipalities with a total of about 60,000 citizens

**Table 2:** Sectors, task division and examples of organizations in the network

Sectors	Tasks	Examples of organizations
1. Center for youth and family	Gatekeeper	Child and youth welfare and healthcare center
2. Municipality	Signaling	Youth care expert team, youth and family team*, school attendance officers, youth/social support/community service/employment/safety/purchase and contracting departments of the municipality
3. Basic social organization	Signaling providing services	Social work, welfare work, disabled support, youth and family support, library, food bank, refugee council
4. Education	Signaling	Care coordinators primary and secondary education
5. General practitioners	Gatekeeper	Child and family doctors
6. Health and prevention	Signaling gatekeeper	Child and youth health care center, infant welfare center
7. Childcare and nursery	Signaling providing services	Pre-school, child day-care center, nursery, after school-care including homework support
8. Specialized youth care	Providing services	Youth mental health care, child and youth care, (forensic) psychiatry, orthopedagogy, psychology, disabled child care
9. Protection and social rehabilitation	Providing services	Youth protection, youth probation officers, juvenile social rehabilitation
10. Safety	Signaling providing services	Police officers responsible for juveniles, protection of child maltreatment, safety houses (crime prevention), public prosecutions department, family and youth court, juvenile prison, child care and protection board, community service supervisor
11. Volunteer organization	Signaling providing services	Village or ward council, social policy advisory council, informal help for family or neighbors, community center, scouting/music/sport/leisure clubs

Note(s): * Youth and family teams also provide support services

**Table 3:** Structure of three networks based on all contacts

	Network I	Network II	Network III
Number of sectors	11	10	11
Number of organizations	135	86	75
Active organizations (%)	135 (100%)	86 (100%)	75 (100%)
Isolates	0	0	0
Number of ties	3368	1728	1950
Density	0.186	0.236	0.351
Average degree centrality	24.95	20.09	26.00
Degree centralization	0.659	0.649	0.666

**Table 4:** Structure of three networks based on client referral contacts

	Network I	Network II	Network III
Number of organizations	135	86	75
Active organizations (%)	132 (98%)	80 (93%)	69 (92%)
Isolates	3	6	6
Number of ties	2102	1026	1056
Density	0.116	0.140	0.190
Average degree centrality	15.57	11.93	14.08
Degree centralization	0.723	0.471	0.415

**Table 5:** Ten organizations with most central position in the client referral networks